# A Worldwide Phylogeography for the Human X Chromosome

**DOI:** 10.1371/journal.pone.0000557

**Published:** 2007-06-27

**Authors:** Simone S. Santos-Lopes, Rinaldo W. Pereira, Ian J. Wilson, Sérgio D.J. Pena

**Affiliations:** 1 Departamento de Bioquímica e Imunologia, Universidade Federal de Minas Gerais, Belo Horizonte, Minas Gerais, Brazil; 2 Programa de Pós Graduação em Ciências Genômicas e Biotecnologia, Catholic University of Brasília (UCB), Brasília, Brazil; 3 Institute of Human Genetics, Newcastle University, Newcastle, United Kingdom; University of Canterbury, New Zealand

## Abstract

**Background:**

We reasoned that by identifying genetic markers on human X chromosome regions where recombination is rare or absent, we should be able to construct X chromosome genealogies analogous to those based on Y chromosome and mitochondrial DNA polymorphisms, with the advantage of providing information about both male and female components of the population.

**Methodology/Principal Findings:**

We identified a 47 Kb interval containing an *Alu* insertion polymorphism (*DXS225*) and four microsatellites in complete linkage disequilibrium in a low recombination rate region of the long arm of the human X chromosome. This haplotype block was studied in 667 males from the HGDP-CEPH Human Genome Diversity Panel. The haplotypic diversity was highest in Africa (0.992±0.0025) and lowest in the Americas (0.839±0.0378), where no insertion alleles of *DXS225* were observed. Africa shared few haplotypes with other geographical areas, while those exhibited significant sharing among themselves. Median joining networks revealed that the African haplotypes were numerous, occupied the periphery of the graph and had low frequency, whereas those from the other continents were few, central and had high frequency. Altogether, our data support a single origin of modern man in Africa and migration to occupy the other continents by serial founder effects. Coalescent analysis permitted estimation of the time of the most recent common ancestor as 182,000 years (56,700–479,000) and the estimated time of the *DXS225 Alu* insertion of 94,400 years (24,300–310,000). These dates are fully compatible with the current widely accepted scenario of the origin of modern mankind in Africa within the last 195,000 years and migration out-of-Africa *circa* 55,000–65,000 years ago.

**Conclusions/Significance:**

A haplotypic block combining an *Alu* insertion polymorphism and four microsatellite markers on the human X chromosome is a useful marker to evaluate genetic diversity of human populations and provides a highly informative tool for evolutionary studies.

## Introduction

Human Y chromosomes are haploid and lack recombination over most of their length. Thus, they are transmitted by males to their male offspring and remain unaltered from generation to generation, establishing patrilineages that remain stable until a mutation supervenes. Human Y chromosomal DNA polymorphisms are consequently paternal lineage markers that have been extremely useful in human evolutionary studies [1].

Since in males the X chromosome is also haploid, determination of haplotypes is straightforward. We reasoned that if we could identify genetic markers on the human X chromosome in regions where recombination is rare or absent, we might be able to study human X chromosome genealogies in an analogous fashion to those based on investigations of Y chromosome and mitochondrial DNA polymorphisms. These X chromosome genealogies would have the interesting peculiarity that in every generation half of the X chromosomes in females and all X chromosomes in males (2/3 of the total) will change sexes [2]. Thus, X chromosome lineages should provide simultaneous information about both the male and female components of the population. This contrasts with Y chromosome genealogies, which examine only patrilineages, and with mtDNA genealogies, which examine only matrilineages. Several authors have emphasized that the history of patrilineages and matrilineages in human populations are diverse [3]. Thus, the comparison of X chromosome genealogies with those of Y chromosomes and mtDNA should be informative of past population history.

With this in mind, we decided to study a region located between Xq13.3 and Xq21.3, with a recombination rate of 0.6 cM/Mb, a low rate when compared with the average X chromosome recombination rate of 1.3 cM/Mb [4]. Within this region we located a young *Alu* element embedded within a *LINE-1* element, which proved to be polymorphic in humans. We recently reported [5] a survey of the worldwide frequency distribution of the new polymorphic *Alu* insertion (named *DXS225*; GDB:11524531) in 677 males from the HGDP-CEPH Human Genome Diversity Panel [6]. All regions of the globe, namely Africa, Middle East, Central Asia, Oceania, Europe and America, showed presence of the *Alu* sequence in polymorphic frequencies, indicating that insertion event took place before the modern human spread from Africa. Further analysis, however, revealed that among the five Amerindian populations in the CEPH panel and two other studied, only the Karitiana showed presence of the *Alu* insertion. The Karitiana are a very small group known to have had contact with European and African descendants in the early 20^th^ century [7] and it is thus most likely that the *Alu* insertion allele was introduced into their gene pool by admixture. Thus, we believe that the *DXS225* is monomorphic in pre-Columbian Amerindians, conceivably because of a founder effect. Because of that, the Karitiana were removed from the analyses in the present article.

In an effort to increase the resolution power of our X-chromosome molecular analysis we searched for and identified seven microsatellites in a 118 Kb region containing the Alu insertion polymorphism. We typed these microsatellites in all 677 male samples of the HGDP-CEPH panel. Here, we report that four of these microsatellites, spanning a 47 Kb interval containing the *DXS225* locus, are in complete linkage disequilibrium, thus providing a hypervariable and highly informative haplotype block for inference about human evolution [8]. The study of the worldwide variation of haplotypes in this region and its exploration using haplotype networks and coalescent analysis provides interesting new knowledge about the population history of humanity after its exodus from Africa.

## Materials and Methods

### Population samples

All unrelated male samples from HGDP-CEPH Human Genome Diversity Cell Line Panel [6] were analyzed in this study. A total of 677 male individuals representing 52 different populations from seven regional groups worldwide (Africa, Europe, Middle East, Central/South Asia, East Asia, Oceania and America). However, as evidence obtained in our previous study with *DXS225* had shown that the Karitiana may have received gene flow from European and/or African populations and also because the group represents a single extended family [7], we removed them from all further analyses. Thus, our final study sample numbered 667 males.

### DNA typing

DNA from each individual was independently typed for the *DXS225 Alu* insertion on X chromosome (Genome Data Base accession number GDB: 11524531) exactly as described elsewhere [5]. As before, the two *DXS225* allelic states were identified as 0 (pre-insertion allele) or 1 (*Alu* insertion allele).

The following seven microsatellites located on Xq21 were also analyzed in all samples: *DXS995, DXS8076, DXS1012, DXS1002, DXS1019, DXS8114 and DXS1050.* The dinucleotide repeat microsatellites *DXS995, DXS8076, DXS1002, DXS8114* and *DXS1050* had been previously mapped to the chosen region by Dib et al. [9] and we used the primers described by them, with exception of the reverse primer of *DXS995* to which was added a tail of ten adenine residues in order to increase the amplicon size and avoid overlap with alleles of the locus *DXS8114* in the multiplex analysis. The pentanucleotide repeat microsatellite *DXS1012* and the dinucleotide microsatellite *DXS1019* were identified using the Tandem Repeats Finder program [10] in the interval 84,261,735 to 84,391,735 of the human X chromosome (GenBank Accession # NT_011651.16). All microsatellite alleles were identified by their repeat numbers. Primers were designed by routine techniques and after verification that the microsatellites were polymorphic, they were registered in the Genome DataBase with accession numbers GDB:11524532 for *DXS1012* and GDB:11524534 for *DXS1019*.

Microsatellites were amplified in multiplex PCR reactions, in a final volume of 10 µl, containing 50 ng of genomic DNA, and separated in multiplex reaction in the capillary automatic sequencer MegaBACE 1000 (GE Healthcare). The results were analyzed using the program Fragment Profile version 1.2 (GE Healthcare).

### Statistical Analyses

The genetic structure of the populations and basic parameters of molecular diversity, including analyses of molecular variance (AMOVA) [11], haplotype frequency, haplotype diversity, haplotype sharing and linkage disequilibrium analyses were calculated using the package *Arlequin* 2.0 [12]. The Product of Approximate Conditionals model of Li and Stephens [13] was used to further investigate the recombination rate over the entire region and over the proposed non-recombining block (*DSX1012, DXS1002 DXS225, DXS1019 and DXS8114*). This method depends on using a fixed value for the scaled population mutation rate, *ϑ* which Li and Stephens [13] call 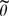. Values for 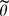 from 10 to 60 were investigated, consistent with known microsatellite mutation rates and effective population size for the X chromosome. While the Li and Stephens [13] model is generally corrected for the number of sites and sequences, we were not interested in a precise estimate of *ρ*, rather we wanted to test whether it was different from zero and thus we did not apply their correction.

Median-joining networks were constructed using the software Network 4.1.0.6 [14] available at www.fluxus-engineering.com.

The program BATWING [15], [16] was used for a genealogical analysis. BATWING uses Markov chain Monte Carlo (MCMC) techniques to sample many reconstructed genealogies proportional to their probability under the coalescent model (for background see Wilson et al. [16]) in a Bayesian framework. These reconstructed population histories depend on models for mutation and the expected genealogical structure and *prior* distributions for parameters of interest. By summarizing the population histories we can see the sorts of population history and ranges of parameters that are consistent with the data in the present.

Further modeling of the population structure is achieved by having a *supertree* that describes each population's history as a sequence of splitting events; this is different to *island* models of structure that assume fixed populations with migrations between them. While the supertree model should not be taken too literally-splitting may take place over many generations and later admixture is always likely–this allows us to take account of the non-random nature of sampling and the correlations between the population histories of individuals within subpopulations.

## Results

### Linkage disequilibrium

We used the *Arlequin* 2.0 program [12] to perform linkage disequilibrium (LD) analyses of the seven microsatellites (*DXS995, DXS8076, DXS1012, DXS1002, DXS1019, DXS8114, DXS1050*) and the *Alu* insertion (*DXS225*) using data from 667 males in the HGDP-CEPH Diversity Panel [6]. According to the March 2006 version of the UCSC Genome Browser (http://genome.ucsc.edu/), the loci are in the order given below and occupy the following positions in contig NT_011651.16 that contains the sequence of the X chromosome: *DXS995* (82,643,697-82,644,081 pb); *DXS8076* (82,665,965-82,666,202 pb); *DXS1012* (85,409,962-85,410,320 pb); *DXS1002* (85,413,714-85,414,062 pb); *DXS225* (85,424,344-85,424,694 pb ); *DXS1019* (85,425,383-85,425,527 pb); *DXS8114* (85,500,625-85,501,030 pb); *DXS1050* (87,160,603-87,160,886 pb).

The linkage disequilibrium test performed by the *Arlequin* 2.0 program is an extension of Fisher exact probability test on contingency tables and the results are reported as *P*-values with standard errors [12]. Obviously, small *P*-values indicate high linkage disequilibrium. As shown in the part below the diagonal of Table 1, we observed linkage disequilibrium for all pairwise tests of markers *DXS1012, DXS1002, DXS225, DXS1019* and *DXS8114* (Table 1). However, no significant linkage disequilibrium was observed between the external loci *DXS995, DXS8076 and DXS1050* (Table 1).

**Table 1:** Pairwise linkage disequilibrium between the seven microsatellites and the polymorphic *Alu* insertion.

	DXS995	DXS8076	***DXS1012***	***DXS1002***	***DXS225***	***DXS1019***	***DXS8114***	DXS1050
**DXS995**	-	0.11	0.17	0.13	0.03	0.06	0.19	0.07
DXS8076	0.920	-	0.14	0.13	0.10	0.12	0.14	0.13
***DXS1012***	0.108	0.160	-	***0.48***	***0.56***	***0.52***	***0.39***	0.10
***DXS1002***	0.622	0.000	***0.000***	-	***0.68***	***0.61***	***0.44***	0.14
***DXS225***	0.912	0.168	***0.000***	***0.000***	-	***0.94***	***0.72***	0.13
***DXS1019***	0.958	0.006	***0.000***	***0.000***	***0.000***	-	***0.64***	0.16
***DXS8114***	0.001	0.000	***0.000***	***0.000***	***0.000***	***0.000***	-	0.19
DXS1050	0.009	0.052	0.069	0.107	0.015	0.010	0.000	-

Below the diagonal are given the *P*-values for rejecting the null hypothesis of free recombination. The standard errors of all *P*-values are less than 0.001. Above the diagonal are displayed the values of D'. The loci that appear to be in complete linkage disequilibrium are shown in bold italics.

Multiallelic D' values [17] are also shown in Table 1, above the diagonal. It should be observed that for the *DXS1012, DXS1002, DXS225, DXS1019* and *DXS8114* block the values go from a high of 0.94 down to 0.39 (Table 1). The problem is that it is difficult to predict theoretically exactly what range of values we would expect for highly variable microsatellites under complete linkage disequilibrium. To ascertain whether our D' values were consistent with zero recombination, we performed a small scale simulation study for four completely linked microsatellites with the same mutation rates as our sample (see below) and a single UEP. The simulations are a standard coalescent with 667 samples, with stepwise mutations for the microsatellites. In Fig. S1 histograms of the minimum, mean (observed mean = 0.6) and maximum D' values seen in 1000 replicates are displayed. Inspection of the histograms reveals that our data are perfectly compatible with absolute linkage disequilibrium.

As an additional test of linkage disequilibrium, the PAC method of recombination rate estimation [13], modified to deal with high mutation rate markers, was used to estimate the recombination rate for the region containing the markers *DXS1012, DXS1002, DXS225, DXS1019 and DXS8114*. The PAC analyses showed no evidence that the population recombination rate, *ρ*, was different from zero for the putative non-recombining sub-block while the entire region had a maximum *ρ* of about 1.5 cm/Mb when 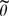 = 40 (further details are shown in Fig. S2).

From the above we conclude that our marker loci *DXS1012, DXS1002, DXS225, DXS1019* and *DXS8114* constitute a non-recombining haplotype block. All subsequent analyses were made using only these markers.

### Haplotypes and their diversity

Among the 667 individuals studied (after removal of the Karitiana) we observed 187 different haplotypes of *DXS1012, DXS1002, DXS225, DXS1019* and *DXS8114.* The number of individuals studied and of haplotypes seen in each of the five major regions is shown in Table 2, together with haplotypic diversity estimates and their standard errors. The haplotypic diversity was highest in Africa (0.992±0.0025) and lowest in the Americas (0.839±0.0378), where no insertion alleles of *DXS225* were observed.

**Table 2:** Number of haplotypes and the haplotypic diversity of the five regions from CEPH panel.

Region	Number of individuals	Number of haplotypes	Haplotype diversity
Africa	98	71	0.992±0.003
East Asia	173	67	0.967±0.005
Eurasia*	342	87	0.953±0.006
Oceania	21	10	0.885±0.047
Americas	33	10	0.839±0.038

* Eurasia encompasses Europe, Middle East and Central Asia.

**Figure 1:**
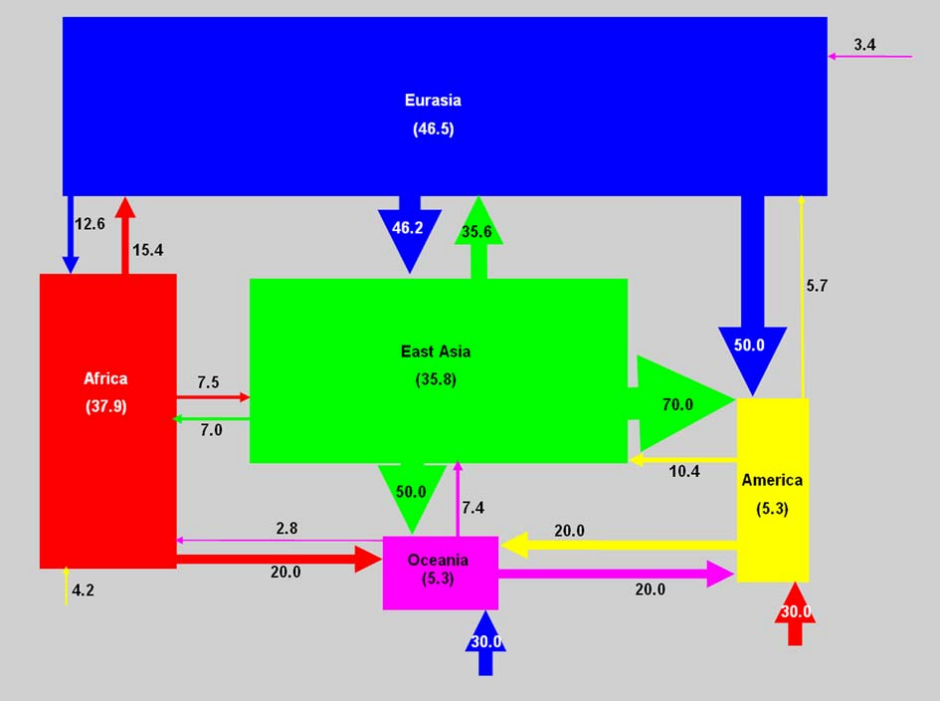
Haplotypes shared among different regions of the world. The area of the rectangles is proportional to the size of the sample from each region. Arrow widths are proportional to the percentage of haplotype sharing from one region to another and the percentages are displayed in the arrows. For instance, 50% of the haplotypes of Oceania are present in East Asia, 20% are present in America, 20% are present in Africa and 30% are present in Eurasia. In contrast only 7.4% of East Asian haplotypes are shared with Oceania. This asymmetry suggests that East Asia is a parental population of Oceania. This figure was inspired by a similar diagram in Conrad et al. [33].

Of the 187 haplotypes encountered, 129 (69.0%) were observed in one single geographical region. Africa contained only 14.7% of the individuals studied, but 44.2% (57/129) of the unique haplotypes. The proportion of shared haplotypes between the different regions is shown in Fig. 1. It is noticeable that Africa shares few haplotypes with the other geographical areas, which in turn display significant sharing among themselves.

A hierarchical analysis of molecular variance (AMOVA) was performed on the haplotype data and is shown in Table 3. Our analysis of genetic variance showed very little genetic structure, with 95.2% within-population, 1.7% among-populations within-regions and 3.1% among-regions components of genetic variance. The same was true for each geographical group, with >95% of the genetic variability occurring within the population level, except for Oceania (only two populations studied; 14.95% among-populations within-regions component) and the Americas (15.29% among-populations within-regions component).

**Table 3:** Analysis of molecular variance (AMOVA) for the X haplotype block*.

Samples	Number of regions	Number of Populations	Variance components (%)		
			Within populations	Among populations within regions	Among regions
World	1	51	96.23	3.77	-
World	5	51	95.22	1.69	3.08
Africa	1	7	98.01	1.99	-
Eurásia*	3	20	98.18	1.06	0.76
East Asia	1	18	100.59	−0.59	-
Oceania	1	2	85.05	14.95	-
America	1	4	84.71	15.29	-

* The haplotype block is composed of *DXS1012, DXS1002, DXS225, DXS1019 and DXS8114*.

** Eurasia encompasses Europe, Middle East and Central Asia.

### Network analysis

**Figure 2:**
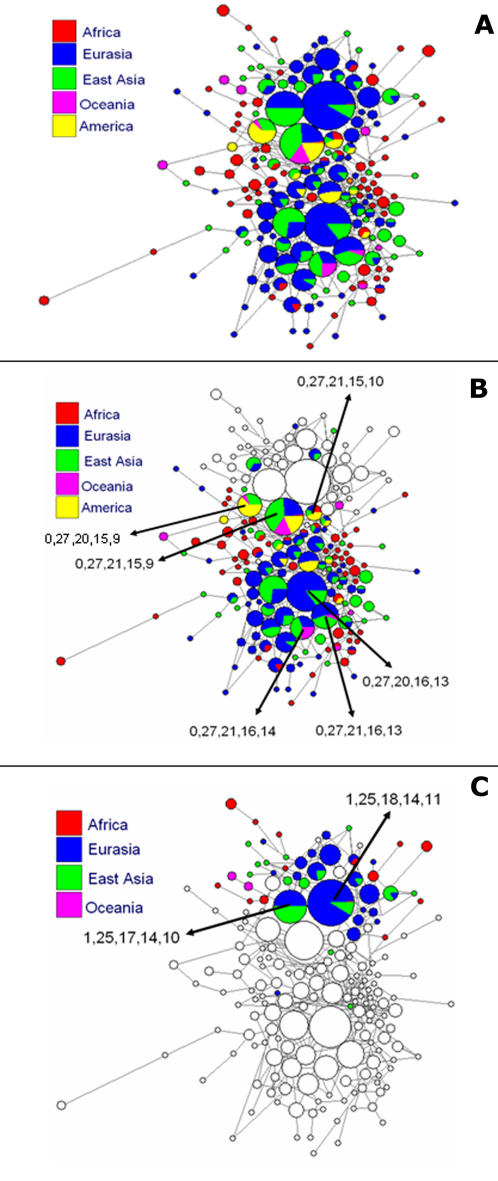
(A) Median joining network of all the haplotypes found in 667 individuals from the HGDP-CEPH Diversity Panel [6], color coded according to region of origin. **(B)** The same median joining network as in (A) with only the haplotypes containing the *DXS225* ^0^ allele shown in color. The most widespread and most common haplotypes are concentrated on two clusters (arrows). **(C)** The same median joining network as in (A) with only the haplotypes containing the *DXS225* ^1^ allele shown in color. The most frequent haplotypes belong to a cluster composed of 1,25,18,14,11 and 1,25,17,14,10 (arrows) and a few others.

Median joining networks (Fig. 2) of haplotypes were drawn using the Network software v.4.201 [14]. In the networks, the 51 populations were color-coded into five groups: Africa, Eurasia, East Asia, Oceania and America. In the global network (Fig. 2a) it was noteworthy the fact that the African haplotypes (red) occupied the periphery of the graph and had low frequency, being often single.

In Fig. 2B we can see the network with only the haplotypes containing the *DXS225* ^0^ allele shown in color. The haplotype 0,27,21,15,9 (*DXS225, DXS1019, DXS8114, DXS1002* and *DXS1012* respectively) is the most common and also the only one seen in all five geographical regions of the world (Fig.2B, arrow). Immediately beside it are two closely related haplotypes also indicated by arrows in Fig.2B: 0,27,21,15,10 (seen in all regions, except Oceania) and 0,27,20,15,9 (seen in East Asia, Oceania and America). The wide geographical spread of these three haplotypes, suggests that one or more of them were among the founder haplotypes in the migrant group that emerged from Africa to populate other continents. A second very common haplotype is 0,27,20,16,13 (arrow in Fig. 2B) seen in Eurasia and East Asia. Clustered with it are two other haplotypes (0,27,21,16,13 and 0,27,21,16,14; Fig. 2B, arrows) both found in Eurasia, East Asia and Oceania. Because of its frequency and wide geographical spread, this family of closely related haplotypes is also a candidate for a second founding effect in the emergence of *Homo sapiens* from Africa.

Moving now to the haplotypes containing the *DXS225* ^1^ allele (Fig.2C) we observe that the most frequent haplotypes belong to a cluster composed of 1,25,18,14,11 and 1,25,17,14,10 (arrows) and a few others. This again suggests a founder effect in the out-of-Africa migration.

### Coalescent analyses

Within each subpopulation, we modeled the genealogy using the coalescent with growth from a constant sized population, as described in Wilson et al. [16]. This model assumes that a small ancestral population (with ancestral population size *N*) grows at a rate α% per generation until it has size Nexp(κ) in the present; this determines how long ago growth started. In this analysis *priors* are needed for the parameters estimated in the model: the mutation rate (*μ*) the growth rate per generation (*α*), the relative sizes of the current and ancestral populations (κ), the ancestral population size (*N*), and parameters that determine the expected shape of the population supertree.

**Figure 3:**
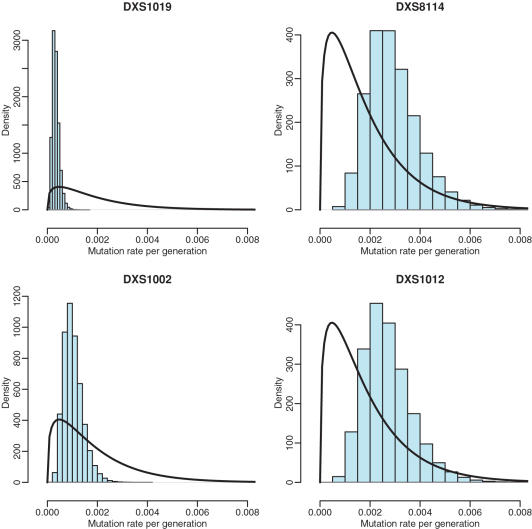
Results from BATWING analysis of the full data set (except Karitiana and Oceania samples). Posteriors histograms (shared bars) and prior densities (solid lines) shown for the mutation rate per generation for the four microsatellites (labels in figure) within the non-recombining region. Posterior histograms are from 200,000 BATWING outputs. Prior density is a gamma(1.35,740.4) for all four microsatellites.

We assumed a stepwise mutation model for the four STR loci: *DXS1019, DXS8114, DXS1002, and DXS1012*, and assumed that *DXS225* is a unique event polymorphism (UEP), i.e., only one mutation has historically occurred at this site. We have used a different mutation rate for each STR, with a gamma distribution with parameters 1.35 and 740.4 to give a mean mutation rate of 0.0018 with a standard deviation of 0.0016. The shape parameter was estimated from the survey of relative mutation rates in Xu et al. [18]. The scale parameter was chosen to give an overall mean mutation rate of approximately 2×10^3^ [8], [19]. All datasets analyzed gave a similar signal for the relative mutation rates of the four loci, with *DXS1019* having an order of magnitude lower mutation rate than *DXS8114* and *DXS1012*. *DXS1002* had an intermediate value. A comparison of the prior and posterior population parameters is shown in Fig. 3.

For the coalescent analysis the populations from Europe, Central Asia and Middle East were treated separately, rather than as a single Eurasian group. Since the sample from Oceania was small it was left out of the analysis, as was the Karitiana as explained above. All analyses used five independent Batwing runs of 42,000 samples with the first 2000 removed from each and with 100 tree rearrangements between each attempted change to the population parameters and only every 200^th^ sample taken. This gave a sample size of 200,000 to construct the empirical posterior distribution. These were very long runs but were used to ensure that the very complex joint model spaces of genealogies and population trees were explored. The results are shown in Table 4. Of special interest are the estimates of the time of the most recent common ancestor (TMRCA) of 182,000 years (95% confidence limits 56,700–479,000) and the estimated time of the *DXS225* Alu insertion of 94,400 years (95% confidence limits 24,300-310,000).

**Table 4:** Posterior means and quantiles for BATWING analysis.

	mean	2.5%	50%	97.5%
Population size of chromosomes, N (×10^−3^)	13.9	4.3	12.8	29.7
Mutation rate μ for DXS1019 (×10^3^)	0.34	0.14	0.32	0.67
Mutation rate μ for DXS8114 (×10^3^)	2.93	1.34	2.78	5.38
Mutation rate μ for DXS1002 (×10^3^)	1.04	0.45	0.98	1.95
Mutation rate μ for DXS1012 (×10^3^)	2.71	1.23	2.57	5.00
TMRCA (kY)	182	56.7	153	479
Time of DXS225 mutation (kY)	94.4	24.3	70.5	310

Data includes populations from Africa, East Asia, Central Asia, Europe, Middle East and America. Populations form Oceania and the Karitiana were excluded from the analysis. Values based on 200,000 samples from the posterior with priors as in Table S1. Note that N is the effective population size of chromosomes. For individuals, assuming equal effective population sizes for males and females, multiply the values by 2/3.

## Discussion

In this study we have used a haplotypic block in X chromosome formed by four microsatellites and one *Alu* insertion to study a worldwide sample of human DNA from the HGDP-CEPH Human Genome Diversity Cell Line Panel [6]. The individuals studied belonged to 52 different populations from seven continental groups (Africa, Europe, Middle East, Central/South Asia, East Asia, Oceania and America). Because Europe, Middle East, Central/South Asia are known to have a very similar genetic structure [20], we decided to pool them into a single group that we called Eurasia. Also, because of evidence indicative that the Karitiana may present admixture from European and/or African sources [5], we elected to exclude them from our analyses.

We reasoned that if we could identify genetic markers on the human X chromosome in regions where recombination is absent, we might be able to unravel human X chromosome genealogies in an analogous fashion to those based on investigations of Y chromosome and mitochondrial DNA polymorphisms. These X chromosome lineages should provide simultaneous information about both the male and female components of the population.

To validate the haplotypic block as a useful non-recombining X-chromosome lineage marker we first determined, using three different statistical approaches that the loci *DXS1012, DXS1002, DXS225, DXS1019* and *DXS8114* were in absolute linkage disequilibrium (Table 1). *DXS225* is a unique event polymorphism characterized by a variable *Alu* insertion that can be seen in all worldwide populations, except in Amerindians [5]. *DXS1019, DXS1002,* and *DXS8114* are dinucleotide repeats and *DXS1012* is a pentanucleotide repeat microsatellite.

Three indirect lines of evidence add support to our idea that recombination between our markers did not happen at all or was a very rare event that should not affect our analyses. First, we obtained no evidence of crossover from European, Chinese and Japanese data for the HapMap [21] (data from HapMap release #22), with the vast majority of SNPs being in complete LD over the entire region (approximately 90% of D' = 1). There were some SNPs not in complete LD, but these were consistent with a small number of gene conversions affecting single SNPs. All LD calculations were performed using Haploview [22] Second, we could not find any evidence from the maps of Myers et al [23] that there were any recombination hotspots within the region delimited by *DXS1012* and *DXS8114*. Finally, the fact that we have observed in the networks a fairly strong founder effect with a small cluster of haplotypes (separated by single step mutations) constituting the vast majority outside Africa provided further evidence for absence of recombination. If recombination had occurred, these founder effects would have dissipated by now.

As expected from the combined variability of the four microsatellites, we have observed 187 different haplotypes among the 667 individuals studied, with a very high haplotypic diversity of 0. 9756±0.0022. Most individuals (477/667; 71.5%) carried the pre-insertion allele at *DXS225* (*DXS225* ^0^) and accounted for a total of 141 haplotypes, a diversity of 0.9718±0.0031. The haplotype diversity for carriers of the insertion allele (*DXS225* ^1^) was 0.8794±0.0167.

The haplotypic diversity of each of the five regions is shown in Table 2. As expected, Africa presented the largest haplotypic diversity. While encompassing only 14.3% of all the individuals studied, Africa contained 44.2% of the unique haplotypes. Accordingly, Africa had small levels of haplotype sharing with other regions (Fig. 1) and in the networks (Fig. 2) African haplotypes mostly occupied the periphery of the graph. Altogether, these observations suggest that the African samples present in the HGDP-CEPH Human Genome Diversity Cell Line Panel embrace only a small portion of the total African haplotypic variability. These data are compatible with the view that modern man emerged in Africa and migrated from that continent to populate all other areas of Earth (reviewed in [24], [25]).

A hierarchical analysis of molecular variance (AMOVA) was performed (Table 3) and revealed negligible amounts of genetic structure. The only exceptions were Oceania and America with, respectively, 14.95% and 15.29% among-populations within-regions component of variation. Since only two populations were studied in the former, it is difficult to ascertain the meaning of this relatively elevated component. As to America, it is well known [26] that the genetic structure of Amerindian populations is characterized by high levels of genetic drift.

Because of the asymmetric nature of haplotype sharing between populations with unequal sample size, its analysis (Fig. 1) reveal genealogical relationships between populations. For instance, our East Asian sample contains 50% of the haplotypes seen in Oceania, but the latter includes only 7% of the haplotypes observed in East Asia. This asymmetry suggests that East Asia has a parental genealogical relationship with Oceania. Likewise, 70% of the haplotypes seen in America are also seen in East Asia while these shared haplotypes make up only 10.4% of the haplotypes of East Asia. In principle this not only suggests that East Asia has a parental genealogical relationship with America (as is known to be the case; see, for instance, [27]), but also that this did not occur too long ago, otherwise there would have been time for much higher levels of mutation-driven haplotypic diversification in Amerindians.

As a consequence of the stepwise nature of microsatellite mutation it is possible to construct useful haplotypic networks (Fig. 2). These show significant different patterns for Africa and the other continents. In the former, as noted above, haplotypes (red) occupy the periphery of the graph and occur at low frequencies, being often single (Fig 2). On the other hand, in Eurasia, East Asia, Oceania and America, the network for carriers of the *DXS225* ^0^ allele was concentrated primarily on two haplotypic clusters (Fig. 2B) while for carriers of the *DXS225* ^1^ allele it was concentrated primarily on one single haplotypic clusters (Fig. 2C). This concentration on few haplotypic clusters outside of Africa can be verified by the estimates of haplotype diversity, which for carriers of the for *DXS225* ^0^ allele were 0.9918±0.0034 in Africa and 0.9623±0.0040 in the other continents. For *DXS225* ^1^ carriers the haplotype diversity was 0.9523±0.0040 in Africa and 0.8549±0.0201 in the other continents. Thus, as expected from the lower frequency of the *DXS225* ^1^ the bottleneck was more severe for carriers of the insertion allele in *DXS225*. All these observations match perfectly the known fact that the migration of modern humanity out-of-Africa was associated with a population size bottleneck and reduction of variability, as has been shown for mitochondrial DNA [28] and several other markers [29].

Prugnolle et al. [30] showed that geographic distance–not genetic distance–from East Africa along likely colonization routes was highly correlated with the genetic diversity of human populations. These and other observations led to an attractive model of human expansion out-of-Africa having occurred by a series of founder effects [31], [32]. Our data are quite in harmony with this serial founder effect model. We first observed a large reduction of haplotypic diversity in the passage from Africa (haplotypic diversity = 0.992±0.003) to East Asia (0.967±0.005), followed by a smaller reduction of haplotypic variability from East Asia to Eurasia (0.953±0.006) and a steeper one from East Asia to Oceania (0.885±0.047). Finally, the peopling of the Americas from East Asia was again accompanied by a significant bottleneck that led to a large reduction of haplotypic diversity (0.839±0.038) and, we believe, to the exclusion of the less frequent *DXS225* insertion allele. Our asymmetric haplotype sharing model (Fig. 1) is completely coherent with this view.

A corollary of the stepwise mutation nature of microsatellite mutations is the fact that we can use microsatellite variability to estimate the timing of evolutionary events. From the coalescent analysis we could estimate the time of the most recent ancestor (TMRCA) as 182,000 years (95% confidence limits 56,700–479,000) for all the individuals studied (Table 4). This is fully compatible with the genetic and paleontological evidence of the origin of modern mankind in Africa within the last 195,000 years [33]. We also estimated (Table 4) that the *Alu* insertion event in DXS225 occurred 94,400 years (95% confidence limits 24,300–310,000). This date is quite consistent with the notion that the Alu insertion occurred in Africa, predating the out-of-Africa migration of man to Asia, which was calculated to have occurred 55,000–65,000 years ago [32], [34].

In conclusion, our results demonstrate that a haplotypic block on the human X chromosome is a useful marker to evaluate genetic diversity of human populations and the combination of an *Alu* insertion polymorphism and microsatellite markers provides a highly informative tool for population and evolutionary studies. Although this is essentially the tale of a single “gene”, similarly to other haplotypes blocks such as the Y chromosome and mitochondrial DNA, it presents a novel, less sex-biased adjunct to studies performed with these traditional markers. Other non-recombining regions in the human genome, as they are identified, can likewise be explored for phylogeographical studies, each one providing a fresh perspective on human evolutionary history. Hopefully, from this multitude of points of view, a coherent picture of the genealogy of the human species will emerge.

## Supporting Information

Table S1 Table of prior means and quantiles for the parameters in the model.
(0.03 MB DOC)

Figure S1 Values for the minimum, mean and maximum values of D' [18] calculated from 10,000 coalescent simulations of 667 individuals each with four completely linked microsatellites (with theta = 4,10,30, and 30 respectively) and one unique event polymorphism (that was selected to have a frequency close to 0.3). The simulations are for a single panmictic population of constant size. Observed values from data are shown by blue arrows.
(0.01 MB EPS)

Figure S2 Scaled approximate log-likelihood curves from the PAC model of Li&Stephens [13] for the entire regions (black lines) and for the proposed non-recombining block of DSX1012, DXS1002 DXS225, DXS1019 and DXS8114 (blue lines) for estimated theta = 10 (dashed lines) and estimated theta = 40 (solid lines). All lines are scaled so that the maximum value is at zero, so allow comparison of lines.
(0.01 MB EPS)
