# Supplementary material for: A Worldwide Phylogeography for the Human X Chromosome
Source: PLoS One. 2007 Jun 27;2(6):e557. doi: 10.1371/journal.pone.0000557 (PMC1891433; doi:10.1371/journal.pone.0000557)
Supplement: Table S1 — Table of prior means and quantiles for the parameters in the model. (0.03 MB DOC) [file pone.0000557.s001.doc]

|  | mean | 2.5% | 50% | 97.5% |
| --- | --- | --- | --- | --- |
| Population size of chromosomes, N (×10^-3^) | 15 | 4.88 | 14 | 30.7 |
| Mutation rate μ (×10^3^) | 1.83 | 0.11 | 1.40 | 5.93 |
| Growth Rate, α (% per generation) | 0.5 | 0.061 | 0.419 | 1.395 |
| Time Since Start of Growth | 51.8 | 5.58 | 27.9 | 220 |
| Relative Population size for Africa | 0.17 | 0.02 | 0.15 | 0.41 |
| First Split (kY) | 37.50 | 961 | 26 | 138 |
| Time of Africa Split (kY) | 87.4 | 2.85 | 68.9 | 274 |
| Last Split (kY) | 187 | 61 | 175 | 384 |
| TMRCA (kY) | 680 | 164 | 558 | 1910 |
